# A cross-country comparison of intensive care physicians’ beliefs about their transfusion behaviour: A qualitative study using the theoretical domains framework

**DOI:** 10.1186/1748-5908-7-93

**Published:** 2012-09-21

**Authors:** Rafat Islam, Alan T Tinmouth, Jill J Francis, Jamie C Brehaut, Jennifer Born, Charlotte Stockton, Simon J Stanworth, Martin P Eccles, Brian H Cuthbertson, Chris Hyde, Jeremy M Grimshaw

**Affiliations:** 1 Centre for Practice-Changing Research, Ottawa Hospital Research Institute, General Campus, Ottawa, Canada; 2 Department of Medicine, University of Ottawa, Ottawa, Canada; 3 Health Services Research Unit, University of Aberdeen, Foresthill, Aberdeen, UK; 4 Department of Epidemiology and Community Medicine, University of Ottawa, Ottawa, Canada; 5 Programme Grant Co-ordinator, University Hospital of South Manchester, Manchester, UK, England; 6 National Health Service Blood & Transplant, Oxford Radcliffe Hospitals, University of Oxford, Oxford, UK, England; 7 Institute of Health and Society, Newcastle University, Newcastle, UK, England; 8 Department of Critical Care Medicine, Sunnybrook Health Sciences Centre, Toronto, Ontario, Canada

## Abstract

**Background:**

Evidence of variations in red blood cell transfusion practices have been reported in a wide range of clinical settings. Parallel studies in Canada and the United Kingdom were designed to explore transfusion behaviour in intensive care physicians. The aim of this paper is three-fold: first, to explore beliefs that influence Canadian intensive care physicians’ transfusion behaviour; second, to systematically select relevant theories and models using the Theoretical Domains Framework (TDF) to inform a future predictive study; and third, to compare its results with the UK study.

**Methods:**

Ten intensive care unit (ICU) physicians throughout Canada were interviewed. Physicians’ responses were coded into theoretical domains, and specific beliefs were generated for each response. Theoretical domains relevant to behaviour change were identified, and specific constructs from the relevant domains were used to select psychological theories. The results from Canada and the United Kingdom were compared.

**Results:**

Seven theoretical domains populated by 31 specific beliefs were identified as relevant to the target behaviour. The domains *Beliefs about capabilities* (confident to not transfuse if patients’ clinical condition is stable), *Beliefs about consequences* (positive beliefs of reducing infection and saving resources and negative beliefs about risking patients’ clinical outcome and potentially more work), *Social influences* (transfusion decision is influenced by team members and patients’ relatives), and *Behavioural regulation* (wide range of approaches to encourage restrictive transfusion) that were identified in the UK study were also relevant in the Canadian context. Three additional domains, *Knowledge* (it requires more evidence to support restrictive transfusion), *Social/professional role and identity* (conflicting beliefs about not adhering to guidelines, referring to evidence, believing restrictive transfusion as professional standard, and believing that guideline is important for other professionals), and *Motivation and goals* (opposing beliefs about the importance of restrictive transfusion and compatibility with other goals), were also identified in this study. Similar to the UK study, the Theory of Planned Behaviour, Social Cognitive Theory, Operant Learning Theory, Action Planning, and Knowledge-Attitude-Behaviour model were identified as potentially relevant theories and models for further study. Personal project analysis was added to the Canadian study to explore the *Motivation and goals* domain in further detail.

**Conclusions:**

A wide range of beliefs was identified by the Canadian ICU physicians as likely to influence their transfusion behaviour. We were able to demonstrate similar though not identical results in a cross-country comparison. Designing targeted behaviour-change interventions based on unique beliefs identified by physicians from two countries are more likely to encourage restrictive transfusion in ICU physicians in respective countries. This needs to be tested in future prospective clinical trials.

## Background

Blood transfusion is an important part of healthcare; when used appropriately, it can save lives and improve patients’ health outcomes*.* However, blood is a scarce and costly resource [1], and there are risks associated with transfusion [2]. Hence, it is important to ensure that blood products are transfused only when appropriate. A widespread deficiency in clinical knowledge about indications and consequences of red blood cell transfusion among general surgeons, orthopedic surgeons, and anaesthesiologists was reported by Salem-Schatz and colleagues [3]. Evidence of variation and suboptimal transfusion practices have been reported for diverse blood products across a wide range of clinical settings (including critical care). Patients in intensive care units (ICUs) receive up to 10% of all red blood cell (RBC) transfusions [4]. The Transfusion Requirements in Critical Care (TRICC) randomised controlled trial suggested that a restrictive transfusion strategy (using a haemoglobin transfusion threshold of 7.0 g/dL) is, at least, equivalent and possibly superior to a more liberal transfusion strategy [5]. Numerous national guidelines support a restrictive transfusion strategy [6-8]. Despite this, observational studies in Canada, Europe, and the United States have reported large variations in transfusion practices across different ICUs [4,9-13]. In these studies, the mean pretransfusion haemoglobin levels were between 7.4 and 8.6 g/dL, higher than the transfusion threshold in the TRICC trial.

A wide variety of interventions to improve transfusion have been used, such as guidelines, education (either group or individual), reminders, and audit and feedback [14,15]. The rationale for the choice of these different interventions was rarely made explicit in studies. Further, the poor quality of the studies evaluating these interventions did not allow for any definitive conclusions about the effectiveness of different interventions or combinations of interventions. As a result, we have a very limited understanding of how to improve transfusion practice.

Since clinical behaviours are performed in complex settings and influenced by multiple socio-environmental factors, changing clinical behaviour by incorporating scientific evidence into practice requires understanding of individual- and institutional-level barriers as an initial step in developing interventions [16]. Using theoretical approaches to identify determinants of behaviour for intervention development increases the likelihood that the interventions will be effective by targeting appropriate mediators of change and identifying suggested mechanisms of change [17-19]. Thus far, a number of psychological theories have been used to investigate healthcare professionals’ behaviour [20-23]. To support greater use of psychological theory to explore factors associated with clinical behaviours, Michie and colleagues proposed a theoretical domains framework derived from 33 commonly used behavioural theories that together had 128 psychological constructs [24]. The resulting structure, referred to as the Theoretical Domains Framework (TDF), identified 12 theoretically distinct domains, each composed of conceptually similar psychological constructs. The TDF can be used to identify perceived determinants of behaviour and barriers to behaviour change to aid selection of specific theories for further testing or to design theory-informed behaviour-change interventions. To explore this, two parallel studies in Canada and the United Kingdom were designed that involved a two-step approach: the first step was designed to assess RBC transfusion behaviour in ICU physicians, using the TDF, that would aid in selecting relevant theories, and the second step would use the selected theories to further investigate the identified beliefs and theoretical domains using a predictive questionnaire study [25]. Healthcare systems and the organisation of transfusion services in both Canada and the United Kingdom are similar. National blood services that promote the appropriate usage of blood and blood products exist in both the countries. The similarities allowed the opportunity to compare transfusion behaviour across two countries by using a similar design and methodology. To maximise comparisons of the results of the UK and Canadian studies, the sample (ICU physicians), the behaviour, the context (adult ICU), and most of the methodology were kept constant between the two studies. Francis *et al.* recently reported on the first step of the UK arm of the study that explored factors influencing UK intensive care physicians’ transfusion behaviour to select theories for predictive questionnaire study [26].

This article is one in a series of articles documenting the development and use of the TDF to advance the science of implementation research [27]. This paper reports on the first step of the two-step approach of the Canadian arm of the study in the original paper and subsequently compares the results of step one of both the countries. Thus, the aim of this paper is three-fold: first, we report the results of the Canadian arm of the study that explored factors perceived to influence ICU physicians’ behaviour for ‘watching and waiting instead of transfusing RBCs in patient with borderline haemoglobin’; second, we select psychological theories in a systematic manner for a future predictive study; and third, we compare the results with the UK study when appropriate. The primary difference between the Canadian and the UK methodology was that interviews were coded by two coders in the Canadian study and by one coder in the UK study. This is the first study to compare factors that may influence ICU physicians’ RBC transfusion behaviours between two health systems by using the TDF.

## Method

### Design

This was an interview study using semi-structured one-on-one interviews, based on the TDF, with ICU physicians in the Canadian setting.

### Participants

A purposive sampling procedure followed by a snowball-sampling technique was used to identify ICU physicians for interviews. Potential participants selected by purposive sampling were carefully identified to reflect variations in practice settings (academic and community) and geographic regions to capture a broad range of beliefs towards RBC transfusion practice. Interview participants from the purposive sampling pool were requested to provide up to three names of intensive care physicians whom they thought differed in their training and practice in transfusion behaviour, a technique known as snowball sampling. Ten ICU physicians (nine males, one female) from academic and community hospitals across Canada participated in the study. Saturation across all the theoretical domains was achieved following 10 interviews.

### Materials

To interview the ICU physicians in Canada, the interview topic guide that was developed for the UK study was utilised. The behaviour for the interview topic guide was centred around the evidence from a landmark study, the TRICC trial, suggesting that a restrictive transfusion strategy is, at least, equivalent and possibly superior to a more liberal transfusion strategy [5]. To avoid the potential problem of bias arising from commencing the interview by referring to the TRICC trail, a vague term, *borderline haemoglobin*, was chosen purposely instead of a particular haemoglobin level from the evidence. At the end of the interviews, the participants were asked about their individual interpretation of borderline haemoglobin. The numbers of questions in the interview topic guide ranged from two to five for each domain; prompts were used when necessary to address the specific constructs within the domains [26]. Following two pilot interviews, minor changes were made to address issues such as clarity, interview length, repetitiveness, etc. A cognitive psychologist (JCB) and a transfusion specialist (ATT) from the research team reviewed the pilot interviews to ensure accurate representation of psychological constructs and adequate coverage of transfusion issues. The final version of the interview topic guide is included (See Additional file 1 for Interview Topic Guide).

### Procedure

A letter explaining the purpose of the study was emailed to ICU physicians who were identified and contacted by the transfusion specialist (ATT) on the research team to ascertain their interest in participating (purposive sampling). After receiving a reply email from a physician suggesting his/her interest to participate in our study, a formal letter of invitation, together with the study information sheet and consent form, were forwarded for further consideration. Upon receipt of a signed consent form, the interviews were arranged and facilitated by a trained interviewer (RI). Most, if not all, intensive care physicians were from centres outside of Ottawa and, therefore, did not have professional relationships with the transfusion specialist (ATT). Additionally, the interviewer (RI) had no ties to the physicians being interviewed. The interviews were completed either by phone or face to face. Each interview was recorded (with approval by the participants) using a digital recorder and lasted between 25 and 67 min (M = 41.9, SD = 14.2). The interview audio files were transcribed verbatim and anonymised. An honorarium of $100 was offered to the participating physicians in appreciation for their expert input and to partially compensate for their time.

### Analysis

Interview transcript analysis involved the following five steps.

1. Coding interview transcripts

To facilitate interview transcript analysis and to ensure consistencies in coding, two coders (RI, JB) independently coded responses from two pilot interviews into theoretical domains and compared results to develop a coding scheme. Thereafter, the coders (RI, JB) independently coded the interviews, guided by the coding scheme. It has been suggested that developing a coding scheme for all researchers involved in descriptive coding for qualitative analysis is an essential step that allows researchers to reach an agreement and avoid subjective bias [28]. During the first six interviews, an iterative process was used to clarify coding differences and to ensure consistency for subsequent analyses. Reliability was calculated for the last four interviews by simple percentage agreement/disagreement to measure consistency in coding within and across domains [29,30]. Complete agreement was reached when two coders identified the same response and allocated it to the same domain, whereas partial agreement was reached when two researchers identified the same response but allocated it to different domains. When coders coded the same response differently, the response was allocated into all domains identified by both coders. Disagreements were resolved by including the opinions of both coders.

2. Generating specific beliefs

Statements describing specific underlying beliefs were generated for each response within each domain by the same two coders (RI, JB) working together. A specific belief was defined as a collection of responses with a similar underlying theme that suggested a problem and/or influence of the belief on the target behaviour [26]. For example, the responses ‘guidelines are just guidelines’, ‘guidelines are not gospel’, and ‘there are no rules about going outside guidelines’ were identified as the same specific belief as ‘I can make my decision outside the guideline’. A frequency count for each belief (to represent the number of participants who mentioned the belief) was calculated across all 10 interviews.

3. Identifying relevant theoretical domains

Domains were judged as likely to be relevant for changing the target behaviour if they contained specific beliefs that might be potential barriers for changing transfusion behaviour and fulfilled the following criteria: (1) relatively high frequency of specific beliefs, (2) presence of conflicting beliefs, and (3) evidence of strong beliefs that may impact on the behaviour. All three criteria were considered concurrently to judge relevance of the domains in terms of influencing target behaviour. Therefore, specific beliefs with lower frequency counts but greater clinical significance were considered in identifying domain relevance. The transfusion specialist (ATT) in the research team helped interpret the importance of the specific beliefs from a clinical perspective. The health psychologist from the research team (JJF) was involved throughout the process to help resolve coding differences, critique specific belief generation, and identify relevant domains.

4. Mapping constructs onto specific beliefs

Following selection of the relevant theoretical domains, the beliefs were analysed for psychological constructs from the domains using the methodology proposed by Francis *et al.* in the UK arm of the RBC transfusion study [26]. Three researchers, a health psychologist (JJF), a cognitive psychologist (JCB), and a knowledge-translation researcher (JMG) independently coded all the specific beliefs onto theoretical constructs from the relevant domains. They were provided with a list of specific beliefs (see Additional file 2) and a list of constructs from the relevant domains (see Additional file 3). Although these researchers belonged to the study team, two (JMG, JCB) were completely removed from data collection and coding. The frequency count of each construct was identified and recorded.

5. Selecting relevant theories and models

Through discussion, the research team identified a set of psychological theories and models that best represented the constructs from the relevant domains for developing a predictive questionnaire, by accounting for all selected theoretical constructs from the list. The list of theories and models that were utilised originally in developing the TDF by Michie *et al.* was used to select the psychological theories and models [24].

### Comparison with the UK study results

The results from this study and the UK study [26] were compared on (1) frequency counts of physicians’ specific beliefs within domains, (2) identified specific beliefs (original and comparable) within domains, (3) identified relevant domains (original and comparable) that were likely to influence ICU physicians’ RBC transfusion behaviour (Table 1), and (4) identified psychological theories for future predictive studies (Table 2). 

**Table 1:** Summary table of specific beliefs elicited from semi-structured interviews with Canadian (n = 10) and UK (n = 11) ICU physicians allocated to the 12 theoretical domains

**Domain**	**Specific beliefs**	**Sample quote from Canadian interviews**	**Frequency in CDN interviews**	**Frequency in the UK interview**
			**n = 10**	**n = 11**
***All domains judged as relevant in Canadian and/or UK study***				
Knowledge*,	**I know about the TRICC Trial and other evidence**	**“Probably the TRICC trial comes up in most frequently” (ICU 5)** **“…it did begin addressing which other studies haven’t, is to make us think more rationally about transfusions and their role and their actual benefit.” (ICU 4)**	**10**	**10**
	More evidence is required to support restrictive transfusion practice	“There is not a ton of evidence out there.” (ICU 1) “There is more lack of evidence than evidence period.” (ICU 2)	6	0
Social/professional role and Identity*,+	I don’t adhere to any guidelines	“I don’t specifically use those guidelines.” (ICU 8)	5	0
	I refer to evidence to guide my practice	“…you might as well go right to the source of the studies instead of somebody else’s interpretation of them.” (ICU 10) “…so you know the major studies that have been out since I trained, you know ten years ago, those would be the main things that I would use.” (ICU 6)	7	0
	**Watching and waiting is part of my professional standard**	**“…if I was transfusing every patient…my colleague would say what are you doing? So yea, there are standards of care…” (ICU 8)** **“…in our group we tend to practice in a similar fashion.” (ICU 6)**	**6**	**4**
	**I don’t feel constrained by guidelines as long as I have a good reason**	**“…I think the guidelines are there as a guidelines, but I don’t feel constrained by them…” (ICU 4)** **“It’s one of the things where if you go outside the parameters you have to explain yourself as to why you are doing it” (ICU 8)**	**6**	**9**
	Guidelines are important for other professionals not me	“The guidelines are excellent for people who do not deal with this clinical question a lot” (ICU 1)	7	0
	Guidelines do not affect my professional autonomy		0	5
	Clinical judgment and experience is superior to guidelines and protocols		0	8
Beliefs about capabilities*,+	I am confident that the ICU team can manage by watching and waiting	“…if it is a borderline case and nothing significantly changes probably I can trust my team to stick with the plan.” (ICU 3) “Very confident, they are excellent here.” (ICU 6)	6	0
	**I am confident provided that the patient is stable and in the ICU**	**“Depending on the situation, if the patient is stable it’s not hard; if they are unstable it is very difficult.” (ICU 2)** **“Sometimes the problem is when they are going to another care unit…when they go out of the ICU they get a blood transfusion.” (ICU 7)**	**5**	**9**
	I am in complete control	“There are a few ICU physicians, they decide what patients get transfused in the ICU, full stop, nobody else decide.” (ICU 8)	3	0
	I am confident to watch and wait	“You know I am very comfortable, I don’t have any problems…”(ICU 7)	8	0
	I am confident most of the time		0	7
Beliefs about consequences*,+	Benefits of watching & waiting: Patients do better in general	“It benefits what I am doing to help patients in general…the greater good of management of patients as a whole.” (ICU 9) “…there is accumulating data that shows patients do better if you minimize the amount of blood that they get.” (ICU 1)	4	0
	**Reduce infection and harms**	**“The benefits are you are avoiding the risks of blood transfusion for the patients, so infection all the long list of complications for blood transfusion.” (ICU 3)**	**10**	**11**
	**It reduces cost and saves resources**	**“Increased availability of blood for other people that need it.” (ICU 5)** **“not to expose the person to the downsides of giving blood and to save money by not giving it.” (ICU 10)**	**7**	**10**
	Disadvantages: **Patient’s condition can deteriorate**	**“…your reserve is significantly less…risks for you are higher if something bad happens to you.” (ICU 2)** **“There could be potential organ compromises.” (ICU 6)**	**8**	**8**
	It is more work	“It is usually more work. It is a hell of a lot easier to just write the order…” (ICU 1)	5	0
Motivation and goals*	**It is important to watch and wait**	**“I think it is important. Very important.” (ICU 5)** **“We feel very strongly about it.” (ICU 8)**	**8**	**7**
	**Not as important as other things**	**“.so yes important, but it is certainly not as important as a lot of other things we do.” (ICU 1)**	**6**	**3**
	It conflicts with other goals	“In the Rivers protocol…they give blood to keep the hematocrit above 30, which is more than the TRICC trial would suggest. So there may be trouble where there is conflicting suggestions of how to manage the person.” (ICU 10) “If they are going to the OR…I probably would transfuse them before they go.” (ICU 5)	7	0
	It is generally compatible to the goals	“Most of the time I think it is not incompatible with other strategies.” (ICU 6)	5	0
Social influences*,+	Colleagues are uncomfortable to watch and wait	“…members of the healthcare team who don’t really understand what you are trying to do and are feeling a bit more uneasy, or a little more anxious.” (ICU 1)	4	0
	Other professionals do not influence me	“…I don’t think they [other team members] influence me because I’ve been in that situation before and it hasn’t really affected my decision” (ICU 3)	7	0
	**Other professionals do influence me**	**“…the cardiologist will in my hospital, (they) would like to have a higher threshold…So they are going to influence me.” (ICU 7)**	**6**	**7**
	**Team working: there are very little disagreement**	**“…in our group we tend to practice in a similar fashion…we try to make decisions that are consistent with the general way that we manage things.” (ICU 6)**	**4**	**11**
	**Patients and family issue influence my practice (Jehovah)**	**“…the patients, like if they were Jehovah Witness or something like that, that would probably encourage [watching and waiting]” (ICU 5)**	**5**	**5**
Behavioural regulation*,+	**Alternatives to transfusion**	**“Well the alternatives are trying to improve the red cell production, so optimizing nutritional support, vitamin levels and iron and the other way is about EPO…but it doesn’t seem to have any other outcome benefit thus far…there isn’t all of data to support that practice…” (ICU 9)** **“I would like to see us take less blood for blood gases and things and prevent iatrogenic anaemia” (ICU 1)**	**6**	**10**
	Protocols/Guidelines/Standard of practice	“If [a policy or protocol] was distributed widely and accepted by the group and reviewed” (ICU 6)	9	0
	Processes to educate health care team	“By emphasizing the issues that are surrounding transfusions and educating residents and house staff they are much more rational in [their] use” (ICU 4)	10	0
	Increase team communication	“I think for the most part, if communication is at a high level, like team communication is at a high level and we will make plans, then we will likely stick to that plan, until the person in charge of the team agrees to a change.” (ICU 3)	4	0
	**Strong evidence to change practice**	**“Maybe if there is a big study that is telling me that it is worse to do the kind of practice I am doing, I might wait for another study, but maybe it will influence me to change my practice” (ICU 7)**	**4**	**10**
	**Audit and feedback**	**“…we took a look at nursing practices around some things, and when they were given their own data, they definitely changed some of the nursing stuff, but we do not do that as a matter of routine. Maybe we should.” (ICU 1)**	**3**	**4**
***Domains judged as not relevant in Canadian and UK study***				
Skills	**Skills needed to watch and wait is not difficult**	**“It is usually fairly easy I think.” (ICU 6)** **“…it is not difficult just to observe and wait.” (ICU 7)**	**8**	**11**
	**Mainly clinical skills are needed**	**“I don’t know if there is any specific skill just an awareness of what the possible consequences… I don’t know about the procedural skills, I don’t think there is anything.” (ICU 6)** **“Just check the haemoglobin and make sure that they are not bleeding and just watch…Doctor and nursing skills.” (ICU 3)**	**5**	**9**
Memory, attention and decision processes	**Patient and clinical factors influence my decision**	**“I think that first and foremost is their age and their co-morbidities and their functional capacity…” (ICU 4)**	**10**	**11**
	Judgment and experience influence my decision	“Your experience, it plays a big role and every patient is different…” (ICU 9) “…if you have any inkling that things are not going well with your haemoglobin of 75 then you should transfuse.” (ICU 2)	4	0
	It is an easy decision	“I think it is one of the easier decisions to make actually in the ICU.” (ICU 8)	6	0
	I usually watch and wait	“That is my default, currently that is the way I practice.” (ICU 4)	7	0
	Need to pay attention to patient’s changing clinical condition and be able to react quickly		0	4
Environmental context and resources	Blood supply and blood quality issues	“Getting the blood from the blood bank can be an issue here.” (ICU 6)	5	0
	Environmental issues do not influence my practice	“If it’s busy, it doesn’t influence [me].” (ICU 4)	8	0
	Staffing issues	“Because of the turnover, particularly with house staff, that it often gets forgotten.” (ICU 4) “The nurses are stretched…the nurses will tell you a million times that they can’t watch Mr. Jones as closely.” (ICU 1)	4	0
	Changes in patient’s clinical status or haemoglobin trends will influence whether I watch and wait		0	9
	The patient’s co-morbidities or pre-existing condition will influence whether or not I watch and wait.		0	9
Emotion	Emotion is not an issue	“Not really no” (ICU 5)	10	0
	**Watching and waiting is not stressful**	**“I think on an overall scale it is low stress compared to the other things that we do.” (ICU 9)**	**9**	**7**
	I might be concerned in some situations about watching and waiting		0	5
Nature of the behaviour	**Frequently come across patients with borderline haemoglobin**	**“It comes up in somebody almost every day.” (ICU 1)**	**8**	**3**
	Using less blood and lower haemoglobin triggers	“We don’t transfuse now until a lower trigger, than we used to.” (ICU 10)	4	0
	Education and learning		0	3

Specific beliefs in bold type elicited in both Canadian and UK study.

* Identified as relevant domain in Canadian study.

+ Identified as relevant domain in the UK study.

**Table 2:** Coding of specific beliefs to identify relevant constructs and theories

**[domain number]** **Relevant domain**	**Specific belief (ICU)**	**Construct** **(Coder A)**	**Construct** **(Coder B)**	**Construct** **(Coder C)**	**Agreement Summary**	**Relevant theories & models**
**[**[1]**] Knowledge**	1. I know about the TRICC Trial and other evidences	Knowledge about scientific rationale	Knowledge about scientific rationale	Knowledge	2/3	KAB model
	**2. More evidence is required to support restrictive transfusion practice**	Knowledge about scientific rationale	Knowledge about scientific rationale	Knowledge	2/3	
**[**[3]**]** **Social/professional role and identity (self-standards)**	**3. I don’t adhere to any guidelines**	Professional identity/boundaries/role	Identity	*(Intention and Goals*[6]*)*	0/3	TPB
	**4. I refer to evidence to guide my practice**	Professional identity/boundaries/role	Identity	Professional role	*2/3*	
	5. Watching and waiting is part of my professional standard	Social/group norms	Identity	Professional role	*0/3*	
	6. I don’t feel constrained by guidelines as long as I have a good reason	Professional identity/boundaries/role	Professional identity/boundaries/role	Professional identity/boundaries/role	*3/3*	
	**7. Guidelines are important for other professionals not me**	Professional identity/boundaries/role, Social/group norms	*behavioural regulation*[11]	Professional identity	*2/3*	
**[**[4]**]Beliefs about capabilities (self-efficacy)**	**8. I am confident that the ICU team can manage by watching & waiting**	Self-confidence/professional confidence	Perceived behavioural control *(team working*[9]*)*	Self-efficacy	*0/3*	TPB & SCT
	9. I am confident provided that the patient is stable and in the ICU	Self-confidence/professional confidence	Control—of behaviour and material and social environment	Self-efficacy, Control—of behaviour and material and social environment	*2/3*	
	**10. I am in complete control to make decision to watch and wait**	Perceived behavioural control	Perceived behavioural control	Perceived behavioural control	*3/3*	
	**11. I am confident to watch and wait**	Self-confidence/professional confidence	Professional confidence	Self-efficacy	*2/3*	
**[**[5]**]Beliefs about consequences (Anticipated outcomes/attitude)**	Benefits of watching & waiting:	Outcome expectancies	Outcome expectancies	Outcome expectancy, Attitude, Consequences	*3/3*	TPB & OLT
	**12. Patients do better in general**					
	13. Reduce infection and harms	Outcome expectancies	Outcome expectancies	Outcome expectancy, Attitude, Consequences	*3/3*	
	14. It reduces cost and saves resources	Outcome expectancies, Incentives/rewards	Outcome expectancies	Outcome expectancy, Attitude, Consequences	*3/3*	
	Disadvantage:	Outcome expectancies	Outcome expectancies, Anticipated regret	Outcome expectancies, Attitude, Consequences	3/3	
	15. Patient’s condition can deteriorate					
	**16. It is more work**	Outcome expectancies, Incentives/rewards	Incentives/rewards	Outcome expectancies, Attitude, Consequences	*2/3*	
**[**[6]**]Motivation and goals (Intention)**	17. It is important to watch and wait	Intention	(more like a belief)	Intention, Certainty of intention	2/3	TPB, SCT &**PPA**
	18. Not as important as other things	Goal priority	Goal priority	Goal priority	3/3	
	**19. It conflicts with other goals**	Goal priority	Goal priority	Goal priority, Certainty of intention	3/3	
	**20. It is generally compatible to the goals**	Goal priority	Goal setting	Goal priority, Certainty of intention	2/3	
**[**[9]**]Social influences (Norms)**	21. Some members of health care team are uncomfortable watching and waiting	Team working	Social comparisons	Social/group norms	0/3	TPB
	22. Other professionals (for example: physicians, surgeons, nurses, residents, fellows) do not influence me	Social/group norms	Group conformity	Social pressure, Subjective norms (*i.e.* the motivation to comply part of SN)	2/3?	
	**23. Other professionals do (for example: clinicians, nurses, physiotherapists, hematologists, blood back staff, non-ICU staff) influence me**	Social/group norms	Group conformity	Social pressure, Subjective norms (*i.e.* the motivation to comply part of SN)	2/3?	
	**24. There is very little disagreement within my health care team**	Group conformity, Team functioning	Group conformity	Group conformity	3/3	
	**25. Patients and family issue influence my practice (for example: Jehovah)**	Social/group norms, Social pressure	Social group norms	Injunctive norms	2/2	
**[**[11]**]Behavioural regulation**	26. Alternatives to transfusing include prescribing vitamins, iron, EPO, nutritional support and taking less blood for testing.	?	Alternatives	Generating alternatives	2/3?	AP & OLT
	27. Widely accepted Protocols or Guidelines or Standard of practice	?	B/F (is this Barriers and facilitators?)	*(Groups norms and group conformity*[9]*)*	0/3	
	**28. Processes to educate health care team**	?	B/F	*(Learning and modelling*[9]*)*	0/3	
	**29. Increasing team communication**	?	B/F	*(Team working*[9]*)*	0/3	
	30. Strong evidence	?	B/F	*(Knowledge*[1]*)*	0/3	
	31. Audit and feedback	?	B/F	Self-monitoring, Feedback	0/3	

Coding of each belief by three independent coders, coder agreement and relevant theories (final column).

[#] - domain number as identified by Michie *et al.*, (2005).

Underlined – the constructs identified by majority of coders.

(*italics*) – constructs identified from other domains.

Specific beliefs in bold type are elicited in Canadian study only. Theory in bold is identified in Canadian study only.

Theories/Models: *KAB* Knowledge-Attitude-Behaviour, *TPB* Theory of Planned Behaviour, *SCT* Social Cognitive Theory, *PPA* Personal Project Approach, *AP* Action Planning component of Action Planning/Coping Planning, *OLT* Operant Learning Theory.

### Ethics

The study was approved by The Ottawa Hospital Research Ethics Board.

## Results

### Interrater agreement

For the last four interviews, the interrater agreement between the two coders for all 12 domains ranged from 33.3% to 100% (Table 3). Complete agreement (both coders identified the same quote and put them in the same domain) was highest (80% and 100%) for the *Skills* and *Emotion* domains; moderate (57–68%) for *Memory, attention, and decision processes*, *Social influences*, *Beliefs about consequences*, *Motivation and goals*, *Social/professional role and identity*, and *Nature of the behaviour* domains; and lowest (33.3–41.2%) for the *Behavioural regulation*, *Beliefs about capabilities*, *Knowledge*, and *Environmental context and resources* domains. A total of 44 beliefs from 12 theoretical domains were identified in our study, as compared to 29 beliefs in the UK study. All beliefs from the UK and Canadian study are presented in Table 1, with the frequency that each belief was mentioned in the Canadian and UK studies noted in the final columns.

**Table 3:** Percent agreement calculated across interviews and domains

**Domains**	**Complete agreement**^**1**^	**Partial agreement**^**2**^
Emotion	100.00%	0.00%
Skills	80.00%	4.17%
Memory, attention and decision processes	67.74%	16.67%
Social influences	66.67%	29.17%
Beliefs about consequences	66.67%	25.00%
Motivation and goals	66.67%	12.50%
Social/professional role and identity	65.12%	37.50%
Nature of the behaviour	57.14%	33.33%
Behavioural regulation	41.18%	29.17%
Beliefs about capabilities	38.89%	37.50%
Knowledge	37.50%	50.00%
Environmental context and resources	33.33%	20.83%
Average	60.07%	24.65%

The data in this table is based on the last four interviews only.

^1^Complete Agreement: Two coders identified the same quote and put them in the same domain.

^2^Partial Agreement: Two coders identified the same quote but put them in different domains.

### Domains judged to be relevant to RBC transfusion in the Canadian study

Theoretical domains that were judged to be relevant to RBC transfusion practice among ICU physicians in the Canadian setting were *Knowledge*, *Social/professional role and identity*, *Beliefs about capabilities*, *Beliefs about consequences*, *Motivation and goals*, *Social influences*, and *Behavioural regulation* (Table 1). A total of 31 specific beliefs from seven relevant domains were identified for further analysis (see below).

All participants were aware of both RBC guidelines and the TRICC trial, but six thought that there was a need for more evidence to guide their practice: ‘There is more lack of evidence than evidence, period’ (ICU 2). Thus, the *Knowledge* domain was identified as potentially relevant. Even though six participants identified the target behaviour as part of their professional role, *Social/professional role and identity* was still identified as a relevant domain because of the diversity of views expressed with regard to adherence to the RBC transfusion guidelines. Five participants reported that they did not adhere to any guideline. Six participants acknowledged not feeling any constraint by guideline recommendations, and seven indicated that guidelines were important for other professionals (but not for them). Seven participants indicated that they would rather refer to the original study and not the guideline to make their decision ‘…you might as well go right to the source of the studies instead of somebody else’s interpretation of them’ (ICU 10).

The majority (n = 8) of the participants were confident about performing the target behaviour, and some (n = 5) elaborated by saying that they felt confident to perform the target behaviour when the patient was in a stable condition and in the ICU. Six participants reported having confidence in their ICU team to perform the target behaviour, and three believed themselves to be in complete control of the decision making. Thus, the ICU physicians’ ability to perform the target behaviour was potentially influenced by numerous factors. This prompted us to select the *Beliefs about capabilities* domain for further investigation.

*Beliefs about consequences* was judged to be a relevant domain because participants identified a number of different factors (benefits and risks) that potentially influenced the target behaviour. Among the perceived benefits, all the participants reported that ‘watching and waiting instead of transfusing RBCs in patients with borderline hemoglobin’ would reduce infection and harms caused by transfusion (n = 10), reduce cost and save resources (n = 7), and improve outcomes (n = 4). Eight participants also reported that the target behaviour could risk deterioration in the patients’ medical condition, and five thought that the decision to watch and wait involved more work (Table 1).

When participants were asked how important they felt it was to perform the target behaviour, eight stated it was important, but six thought that it was not as important as other aspects of patient care in the ICU. Seven participants thought that performing the target behaviour could conflict with other specific goals when treating ICU patients, but five participants thought that it was generally compatible with other goals. Thus, variability in the views among participants and sometimes conflicting viewpoints expressed within a participant resulted in the selection of the *Motivation and goals* domain as relevant.

The *Social influences* domain identifies whether other members of the clinical team (other physicians, nurses, and residents) and patients’ relatives may influence physicians’ transfusion decisions. Seven participants indicated that other professionals from the clinical team did not influence their decision to perform the target behaviour. Six participants indicated that other professionals from the clinical team had an impact on their behaviour; however, they identified different team members (*i.e.*, residents, fellows, nurses, physicians from the same specialty as well as other specialty). Four participants indicated that the decision to perform the target behaviour was often made as a team, and five stated that decisions took account of the opinion of the patients’ relatives. Four participants reported that colleagues were sometimes uncomfortable with the final decision about watching and waiting instead of transfusing RBCs. Several, sometimes contradictory (on one occasion within the same interview), beliefs emerged in the *Social influences* domain. Thus, the *Social influences* domain was thought to influence transfusion behaviour and was identified as relevant.

The *Behavioural regulation* domain was judged to be relevant because respondents identified numerous views (Table 1) on how to encourage ICU physicians to perform the target behaviour, such as educating the healthcare team, using alternative approaches to improve patient care, distributing guidelines/protocols, increasing team communication, producing stronger evidence to change practice, and introducing audit and feedback.

### Domains judged to be not relevant to RBC transfusion in the Canadian study

Five theoretical domains representing 13 specific beliefs were reported as not relevant to changing the target behaviour in ICU physicians in the Canadian setting. These were *Skills*; *Memory, attention, and decision processes*; *Environmental context and resources*; *Emotion*; and *Nature of the behaviour* (Table 1).

The *Skills* domain was not identified as problematic as participants repeatedly reported that the target behaviour required only clinical knowledge and not procedural skills: ‘I don’t know if there is any specific skill, just an awareness of what the possible consequences… I don’t know about the procedural skills, I don’t think there is anything’ (ICU 6). They further stated that the skills were not difficult to perform: ‘…it is not difficult just to observe and wait’ (ICU 7).

The majority of the participants referred to various factors, such as their own clinical judgement and experience, patient factors, and clinical conditions, that they considered consistently in the process of making the decision to perform the target behaviour: ‘…if you have any inkling that things are not going well with your haemoglobin of 7.5 g/dL, then you should transfuse’ (ICU 2). They also mentioned that the target behaviour reflected their usual practice and was an easy decision to make. All of this suggested that it was not a difficult decision for them to make, and more importantly, forgetting to perform the target behaviour was not a concern for this group. Therefore, *Memory, attention, and decision processes* was identified as a domain that was not problematic in performing the target behaviour by the participants.

Participants mentioned that the decision to perform the target behaviour was never influenced by environmental factors, resource issues, or time constraints *(Environmental context and resources)*. The ICU setting in particular was the reason why the participants did not experience any constraints from the environment or resource perspective: ‘I think it is one of the easier decisions to make actually in the ICU’ (ICU 8) and ‘If it’s busy, it doesn’t influence [me]’ (ICU 4)*.* Almost every participant reported that in performing the target behaviour, their own emotion never affected their decision.

The *Nature of the behaviour* domain was not selected as relevant for performing the target behaviour because responses in this domain revealed the characteristics of the behaviour in question. The majority of the participants (n = 8) cared for patients with borderline haemoglobin in the ICU almost every day, and four elaborated on their recent transfusion practice, which included a lower haemoglobin trigger and transfusion of less blood in managing such patients.

### Identification of theoretical constructs in the Canadian study

All three researchers or two out of three researchers agreed when mapping the specific beliefs onto theoretical constructs for 22 (71%) beliefs, whereas there was zero agreement on nine beliefs (29%) (Table 2). Researchers discussed their disagreements to reach a consensus and finally mapped the individual theoretical constructs onto relevant theories. The theory-mapping exercise identified the Theory of Planned Behaviour (TPB) [31], Social Cognitive Theory (SCT) [32], Operant Learning Theory (OLT) [33], Action Planning component of the Action Planning/Coping Planning Theory [34], and Personal Project Approach (PPA) [35] as potentially relevant approaches for the predictive study at a later date (Table 2). In addition, they identified the Knowledge-Attitude-Behaviour (K-A-B) [36] intuitive model to include in the study. *Beliefs about consequences*, *Social/professional role and identity*, *Beliefs about capabilities*, and *Social influences* domains were represented by the TPB; the *Beliefs about capabilities* domain was also represented by the SCT; the *Beliefs about consequences* and *Behavioural regulation* domains together were addressed by the OLT as a possible reward system for influencing behaviour; the *Behavioural regulation* domain was also represented by the Action Planning component of the Action Planning/Coping Planning Theory; and the *Motivation and goals* domain was represented by the TPB, SCT, and additionally by the PPA. *Knowledge* was part of the intuitive K-A-B model.

### Comparisons between Canadian and the UK studies

In the Canadian study, the researchers identified 44 specific beliefs, as compared to 29 specific beliefs in the UK study. Comparing the specific beliefs, there were 20 that were common to both the Canadian and UK study, whereas 24 were identified only in the Canadian study and 7 were identified only in the UK study (Table 1). Canadian ICU physicians identified 31 specific beliefs from seven relevant theoretical domains compared to 11 specific beliefs from four theoretical domains identified by the UK ICU physicians (Table 1). Only the *Skills* domain had the same specific beliefs identified in both the countries. Three additional domains (*Knowledge*, *Social/professional role and identity*, and *Motivation and goals*) were identified as potentially relevant to the Canadian study that were not identified in the UK study. The *Knowledge* and *Social/professional role and identity* domains were addressed by the K-A-B intuitive model and subjective norm construct of the TPB, respectively [31]. Since, motivation has been identified as a precursor of intention in the TPB and goals as a conceptually similar idea to proximal goals in the SCT, the motivation and goals components from the *Motivation and goals* domain were effectively addressed by the TPB and SCT. Since in the context of behavioural psychology the concept of personal goals largely overlaps with the goal-directed actions in context, the primary concept of the personal project [35], PPA was added to the list of theories that were selected for the UK study to explore the *Motivation and goals* domain in further detail.

Among the most prominent differences in our study as compared to the UK study was the wide range of variability in physicians’ perceptions with regard to adherence to guidelines (*Social/professional role and identity*), which was not present in the UK study (Table 1). These were not unexpected findings because Canadian participants also expressed greater concern about the inadequate evidence in the transfusion literature (*Knowledge)* and, therefore, would not be expected to have confidence in guidelines perceived to not be supported by strong evidence. The Canadian arm of the study also identified beliefs in the *Social influences* domain (including *colleagues to whom I am handing over at the end of a shift* and *patients’ family/relatives*) that were not elicited in the UK study but that were perceived to influence the participant physicians’ transfusion behaviour in the Canadian sample. Unlike the UK study, where most participants agreed that the target behaviour was important, the ICU physicians in the Canadian study were divided in their opinion about the relative importance of the target behaviour (*Motivation and goals*). Some of the Canadian physicians reported that performing the target behaviour was important and compatible with other ICU treatments, while others thought it was not as important and conflicted with other tasks in the ICU.

## Discussion

### Summary of findings

The present study identified a range of beliefs that may influence transfusion practice among the ICU physicians in the Canadian setting and systematically selected psychological theories for future predictive study, followed by comparing results with the sister study from the United Kingdom. Drawing on the TDF, beliefs identified in the Canadian study were clustered into the domains *Knowledge*, *Social/professional role and identity*, *Beliefs about capabilities*, *Beliefs about consequences*, *Motivation and goals*, *Social influences*, and *Behavioural regulation*. Psychological constructs from these relevant domains were mapped to select TPB, SCT, OLT, Action Planning component from Action Planning/Coping Planning, PPA, and an intuitive model of K-A-B as the most applicable approaches for designing a predictive questionnaire for further investigation of transfusion behaviour in ICU physicians.

The *Knowledge*, *Social/professional role and identity*, and *Motivation and goals* domains were not identified in the UK study. These newly identified relevant domains were generally addressed by the theories and models already selected for the UK study. However, PPA was added in the Canadian study to explore further the goals component from the *Motivation and goals* domain. A greater number of specific beliefs were identified in the relevant domains in the Canadian study (31) compared to the UK (11). This difference in number of specific beliefs between two countries could be because these domains were elaborated to a greater extent among ICU physicians in Canada than in the United Kingdom or possibly merely a function of the analytic style of the coders. In the absence of clear decision rules for comparing qualitative studies, results from two countries were compared based on specific beliefs, relevant domains, and psychological theories.

Since we employed a nearly identical methodology in the Canadian and UK arms of the study, we are able to examine the similarities and differences in factors that may influence transfusion behaviour among ICU physicians in two countries as elicited by the TDF. The identification of four common domains in the two geographically separated countries suggests that there are many common factors that could influence transfusion practice. These similarities are not unexpected given that both countries have advanced medical systems with similar resources and technology. The Canadian medical profession and system share similar roots with the United Kingdom, and with the advances in information technology, ICU physicians are exposed to the same journals and studies.

The differences in domains identified as potentially relevant in the Canadian and the UK arms could represent either true differences in the domains that are perceived to influence transfusion practice by ICU physicians in the two countries studied or variability in interview coding and domain interpretation when using the TDF for content analysis of the interviews. While there are many similarities in the Canadian and UK healthcare systems, they are not identical. There remain important differences in education/training, resources, hospitals, and the general structure of the healthcare system itself that potentially can influence physician behaviour and, thus, contribute to differences in the specific beliefs and domains identified. We believe that in the Canadian arm of the study, data analysis was rigorous due to inclusion of a second coder to code the interviews. However, we also observed marked variability in the interrater reliability among some domains. Variations in the interrater reliability may suggest a lack of clarity among the coders about the underlying concepts of the domains. In the absence of a domain definition, domains with higher interrater agreement suggest that the constructs within a domain are cohesive, resulting in a consistent interpretation of the domain concept by the coders, whereas lower interrater agreements suggest variability in domain interpretation. While the domains with poor agreement between the coders were more likely to be identified as relevant, this was unlikely to be related to the coding agreement, as domain relevance was determined by other factors (*i.e.*, likelihood of beliefs being significant barriers to transfusion behaviour).

### Strengths of the study

This study has several strengths. It is the first study to explore ICU physicians’ beliefs about their transfusion practices in two countries using similar methods. Secondly, we used the TDF to explore and analyse factors that are likely to influence ICU physicians’ transfusion practice. Using this framework added substantial strength to our study because it is composed of theoretically derived domains based on a comprehensive list of behavioural theories that are commonly used in health psychology. This allowed us to identify potentially relevant theoretically derived domains and to select a set of relevant theories to investigate the target behaviour in depth at a later date. Thirdly, utilising nearly identical methods to examine transfusion behaviour in two countries allowed us to compare the results from two studies. Finally, given the goal of identifying all potentially relevant domains, the data analysis by two coders utilising a common coding scheme in the Canadian study made the methods robust. The coding scheme helped the coders (RI, JB) to achieve greater agreement and reduce individual interpretations of the content of interview transcripts.

### Strengths in relation to other studies

In 1990, Salem-Schatz and colleagues reported a study of clinical and nonclinical factors influencing physicians’ transfusion practice in a face-to-face survey of 122 general surgeons, orthopaedic surgeons, and anaesthesiologist in three hospitals [3]. The study reported widespread deficiency in clinical knowledge about indications and consequences of transfusion. In that study, fewer than half of the physicians surveyed could correctly estimate each transfusion risk, only 31% responded correctly to a set of four questions regarding transfusion indications, and attending physicians routinely had lower knowledge scores compared to residents. The measure of self-confidence in the study participants was negatively associated with the summary knowledge score. In our interview study, knowledge was not explored with specific questions as in the study conducted by Salem-Schatz *et al.* In contrast, we covered a broader range of domains that were likely to influence physicians’ transfusion behaviour in a complex clinical setting. Clinical practice is likely to be influenced by a wide array of factors, where knowledge is just one component. Ignoring physicians’ perception about the multitude of factors likely to influence their transfusion behaviour could potentially be a reason for an ineffective behaviour-change intervention.

### Limitations of the study

While this study identified a variety of factors that may influence the ICU physicians’ transfusion behaviour, there were several limitations that we experienced in using the TDF for data analysis and relevant theory selection. Lack of clarity in the definitions of the theoretical domains proved to be a major obstacle, especially for the Canadian study, which utilised two coders to code interviews independently. In an attempt to resolve the disagreements, we often referred back to the psychological constructs and sample questions proposed by the framework to help generate working definitions for each domain. This, however, was challenging when some constructs were found in more than one domain. Individual analytic style was another possible limitation in using the TDF. This may have been a reason why numbers of specific beliefs identified in the Canadian study were higher compared to the UK study. Having a defined domain structure and a shared coding scheme for interview content analysis for all the coders would have likely reduced the subjectivity in interpretation of interview data. The sampling technique may have carried a risk of bias by recruiting a group of colleagues who may have similar practices and opinions. In order to avoid this, participants were requested to identify additional ICU physicians who they thought differed in training and practice in transfusion behaviour. Subsequently, our results suggest that there were differing opinions about transfusion behaviour among the study participants. There was a concern whether the selection of relevant theories was going to be influenced when coders mapped specific beliefs onto different constructs within one domain. Further research is needed to explore the subtle differences between constructs within a domain to improve the objectivity of theory selection.

## Conclusions

Clinical evidence with regard to transfusion practice in an ICU recommends watching and waiting instead of transfusing patients with borderline haemoglobin, a practice that currently occurs at a lower rate than recommended. Interventions to change clinical practice require an understanding of the behaviour that is likely to be influenced by a variety of factors. To investigate factors influencing transfusion behaviour, a two-step approach was taken: the first step consisted of interviews to assess factors influencing intensive care physicians’ transfusion behaviour and to facilitate the selection of relevant theories and the second step consisted of a theory-based predictive questionnaire study. In the first step, the use of TDF with replicating methods allowed us to examine such factors and identify consistent patterns that might influence physicians in Canada and the United Kingdom, through the two arms of our study. The ‘theoretical domains’ approach utilised in the first step also enabled us to identify domains likely to influence ICU physicians’ transfusion behaviour and subsequently a set of theories, which led to the second step of the study to investigate the behaviour in two countries in theory-based predictive studies using questionnaires. The process of identifying theories based on a theoretical framework was an attempt to make theory selection more systematic. The planned predictive studies based on psychological constructs from the identified theories will provide a rationale for designing theory-driven targeted interventions to change transfusion practice among ICU physicians. Monitoring and improving blood product utilisation is an important day-to-day aspect of transfusion medicine [14-37], but the approaches used to date have not been adequately studied. As a result, little is known about the absolute or relative effectiveness of the different approaches that are currently used to improve transfusion practice. Mapping of theoretical domains likely to influence transfusion behaviour will guide selection of behaviour-change techniques for designing theory-driven interventions and elucidate the factors that can influence transfusion practice. Designing interventions by targeting specific psychological constructs and adopting appropriate behaviour-change techniques should advance physician behaviour-change research and the optimisation of transfusion practice.

## Competing interests

The authors declare that they have no competing interests.

## Authors’ contributions

ATT, SJS, CH, MPE, and JMG conceived the study and acquired funding. RI conducted the interviews and, with JB, conducted data analysis and interpretation. RI drafted the manuscript with significant contributions from ATT, JMG, and JJF. JJF, JMG, and JCB were theoretical and methodological advisers. All authors advised on clinical and methodological issues, provided ongoing critique, and approved the final version of the manuscript.

## Supplementary Material

Additional file 1 Interview Topic Guide. Click here for file

Additional file 2 **GUIDANCE: Please read the list of constructs (from Michie*et al.*, 2005) and each of the following 31 statements in the “Specific belief” column.** Assign a construct to each specific belief and write the construct name in the final column. Click here for file

Additional file 3 Domains. Click here for file
