# Supplementary material for: A cross-country comparison of intensive care physicians’ beliefs about their transfusion behaviour: A qualitative study using the theoretical domains framework
Source: Implement Sci. 2012 Sep 21;7:93. doi: 10.1186/1748-5908-7-93 (PMC3527303; doi:10.1186/1748-5908-7-93)
Supplement: Additional file 1 — Interview Topic Guide. [file 1748-5908-7-93-S1.docx]

**Additional File 1: Interview Topic Guide**

**IMPLEMENTATION RESEARCH IN TRANSFUSION MEDICINE: USING THEORETICAL CONTRUCTS TO INFORM AND GUIDE THE SELECTION OF INTERVENTIONS TO CHANGE TRANSFUSION PRACTICE**

***Interview Guide for Semi Structured Interview***

Agenda setting, timings (40-50 mins, 12 sub-sections 2-3mins long) consent, being recorded etc.

**Introduction:** Thanks for agreeing to participate in this interview. “The general aim of the study is to help us understand more about transfusion practice in **Intensive Care Unit** patients. I want to do this by trying to figure out how you think when you’re making the decision about transfusing patients, by getting inside your head as it were. Some of these questions might be quite new to you, so I’d like you to give them some thought and answer frankly.”

We are actually interested in several different theories, covering a number of domains, and therefore, some questions may be similar.

How does that sound?

Are you ready to get started?

“Certain studies have driven our research, one of which is the TRICC trial which is a Canadian ICU study, led by Paul Hébert & colleagues, about different transfusion triggers.”

1. Are you aware of this study? ………………………………………

2. What were your thoughts about it? ………………………………………

(Prompt: Good points; Bad points)

3. Has it influenced your clinical practice? ……………………………

Would you like me to leave you a copy of the paper? [only if know nothing about the trial]

“As I said before, the evidence we have just talked about has driven the current study. As a result, I would like to go through the rest of the interview talking about your clinical practice in light of this evidence.

I am interested to hear your thoughts about how you might:”
**manage a patient with borderline Hb** **by watching & waiting instead of transfusing RBCs.**

When I say borderline Hb, I’m thinking about those patients when the decision about transfusing might be difficult (not those you’d def trans or those you’d def not trans), you know, where there’s a bit of a grey area…

4. What would you consider borderline Hb? …………………………

**A. KNOWLEDGE**

“We have talked about some of the evidences, I’d also like to find out about your knowledge and use of guidelines:”

5. Do you use any guidelines (to inform your transfusion practice)?

(Prompt: If so, which ones? CMAJ, Bloody Easy, CBS guideline, Discipline specific etc

Is there any reason that you do you not use guidelines? Do you think guidelines are a waste of time?)

6. How do you use the guidelines in your daily clinical practice, i.e. what do you actually, physically do? ………………………………

(prompt: behaviour – do you ever read or refer back to the guidelines to check if a

behaviour you performed was guideline compliant?)

7. How important do you think the guidelines are? ………………

(Prompt: who do you think they are important for?)

8. Are you happy with the way the guidelines you use reflect the evidence? ……..

(Prompt: do the guidelines need updating? What other evidence are you aware of, or do you use?)

9. What other evidence are you aware of or do you use?

**B. SOCIAL/PROFESSIONAL ROLE & IDENTITY**

10. Do you sometimes feel constrained by guidelines?.............................

What about protocols?..............................................................................

(Prompt: How does this affect your professional autonomy?)

11. Is there anything else about your professional role that influences how you manage patients with borderline Hb by watching & waiting instead of transfusing RBCs? …………..

(Prompt: is this something you are trained to do; consensus in your profession; acceptance by the peers; standard of practice)

**Set the scene** re: asking about “**managing a patient with borderline Hb by watching & waiting instead of transfusing RBCs**” – I’d like you to think about that for a moment…

**C. SOCIAL INFLUENCES**

12. Would any other team members influence whether or not you manage patients with borderline Hb by watching & waiting instead of transfusing RBCs? ………………

(Prompt: who else; Other clinicians; medical staff including nurses and residents/fellows; relatives; CBS; in what circumstances)

13. How might the views of other team members affect you managing a patient with borderline Hb by watching & waiting?.........

**D. BEHAVIOURAL REGULATION**

14. If you wanted to change your own transfusion practice, how would you do this? ………………………………………………………

(Prompt: Can you think of any recent example? What did you do differently then?)

15. Are there procedures or ways of working that might encourage you to manage a patient with borderline Hb by watching & waiting instead of transfusing RBCs?............................. (Prompt: a set protocol or policy, increasing monitoring)

16. If you decided to manage a patient with borderline Hb by watching & waiting*,* how confident are you that your team can carry this out?………………

**E. NATURE OF THE BEHAVIOUR**

17. In ICU, how often do you come across patients with a borderline Hb?

“The evidence from research suggests that transfusion practice is variable. However, there is evidence to support a restrictive transfusion practice. With that in mind, in terms of **aiming to transfuse less**:”

18. What might need to be done differently to watch and wait instead of transfusing RBCs among patients with borderline Hb? …………………………

(Prompts: What would you do differently? Who needs to do what differently

when, where, how, how often and with whom? Educational/discussion session?)

**F. SKILLS**

19. How easy or difficult would it be to manage a patient with borderline Hb by watching & waiting instead of transfusing RBCs? ..

20. What skills are required to manage a patient with borderline Hb by watching & waiting? ………………………………………………

(knowledge of evidence, discussion about potential harms and benefits)

**G. BELIEFS ABOUT CAPABILITIES**

21. How confident are you about managing a patient with borderline Hb by watching & waiting instead of transfusing RBC,s despite any difficulties? ……………………………

22. What problems/difficulties do you think you might encounter in managing a patient with borderline Hb by watching & waiting? (people with different opinion)

23. What would help you overcome these problems/difficulties? (communication) …

**H. ENVIRONMENTAL CONTEXT & RESOURCES**

24. In what way is managing a patient with borderline Hb by watching & waiting instead of transfusing RBCs affected by different clinical/environmental situations? situations other than what you have already described; situations such as, whether the unit is busy, whether to attend a more urgent clinical issue, how much time to get the RBCs to the unit, cost issue) **………………………………………………………………..**

25. Are there any competing tasks or time constraints that might influence whether you might manage a patient with borderline Hb by watching & waiting? ……………………………………………………

**I. BELIEFS ABOUT CONSEQUENCES**

26. What are the benefits of managing a patient with borderline Hb by watching & waiting instead of transfusing RBCs? ………………

(Prompt: to yourself, to the patients i.e. what harms might be avoided?, colleagues,

healthcare organization [CBS] – positive & negative, long-term & short-term,

financial)

27. What are the disadvantages of managing a patient with borderline Hb by watching & waiting? ………………………………

(Prompt: to yourself, to the patients i.e. what harms might there be? colleagues, healthcare

Organisation [CBS] – positive & negative, long-term & short-term, financial)

28. Are there any incentives to encourage managing a patient with borderline Hb by watching & waiting instead of transfusing RBCs? …………………………………………...

(Prompt: the team gets recognized/gold star at the end of the week for reducing blood use)

**J. MOTIVATION AND GOALS**

29. How important do you feel it is to manage a patient with borderline Hb by watching & waiting instead of transfusing RBCs? …………

(prompt: how high is the priority in relation to other behaviours required to treat the patient)

30. Would the goal of managing a patient with borderline Hb by watching & waiting be incompatible with achieving some other objective? (anything else that you want to do or achieve that might interfere with this) ………………………………………

(Prompt: slowing time to recovery or discharge)

**K. MEMORY, ATTENTION AND DECISION PROCESSES**

31. What thought processes might guide your decision to manage a patient with borderline Hb by watching & waiting?

(Thrust on thinking; Prompt: thinking about xyz factors some could be clinical others: relatives’ preferences, uncertainty about the evidence, time until the patient is discharged, how ill the patient looks)

Is this something you would have to think a lot? (Prompt: Was it an easy or a difficult decision)

32. In what situations, if any, might it be difficult for you to think of alternatives to transfusing?

(Prompt: other than the clinical scenarios that you have already mentioned)

33. Is managing a patient with borderline Hb by watching & waiting something you would usually do?

**L. EMOTION**

34. Can you think of situations in which you would be worried about watching and waiting instead of transfusing? …………………………

35. How would it influence your work stress to manage a patient with borderline Hb by watching & waiting? …………………………

**In Conclusion:**

That’s all the questions I have for you, has anything occurred to you about this topic that we haven’t asked about?......................................

Overall, what was your thought about the interview?
