# Supplementary material for: A cross-country comparison of intensive care physicians’ beliefs about their transfusion behaviour: A qualitative study using the theoretical domains framework
Source: Implement Sci. 2012 Sep 21;7:93. doi: 10.1186/1748-5908-7-93 (PMC3527303; doi:10.1186/1748-5908-7-93)
Supplement: Additional file 2 — GUIDANCE: Please read the list of constructs (from Michieet al., 2005) and each of the following 31 statements in the “Specific belief” column. Assign a construct to each specific belief and write the construct name in the final column. [file 1748-5908-7-93-S2.docx]

**Additional File 2:**

GUIDANCE: Please read the list of constructs (from Michie et al., 2005) and each of the following 31 statements in the “Specific belief” column. Assign a construct to each specific belief and write the construct name in the final column.

| **Relevant Domain** | **Specific Belief (ICU)** | **Construct**  **(please fill in this column)** |
| --- | --- | --- |
| **(1) Knowledge** | 1. I know about the TRICC Trial and other evidences |  |
|  | 2. More evidence is required to support restrictive transfusion practice |  |
| **(3) Social/ professional role and identity (self-standards)** | 3. I don’t adhere to any guidelines |  |
|  | 4. I refer to evidence to guide my practice |  |
|  | 5. Watching and waiting is part of my professional standard |  |
|  | 6. I don’t feel constrained by guidelines as long as I have a good reason |  |
|  | 7. Guidelines are important for other professionals not me |  |
| **(4) Beliefs about capabilities (self-efficacy)** | 8. I am confident that the ICU team can manage by watching & waiting |  |
|  | 9. I am confident provided that the patient is stable and in the ICU |  |
|  | 10. I am in complete control to make decision to watch and wait |  |
|  | 11. I am confident to watch and wait |  |
| **(5) Beliefs about consequences (Anticipated outcomes/attitude)** | Benefits of watching & waiting:  12. Patients do better in general |  |
|  | 13. Reduce infection and harms |  |
|  | 14. It reduces cost and saves resources |  |
|  | Disadvantages:  15. Patient’s condition can deteriorate |  |
|  | 16. It is more work |  |
| **(6) Motivation and goals (Intention)** | 17. It is important to watch and wait |  |
|  | 18. Not as important as other things |  |
|  | 19. It conflicts with other goals |  |
|  | 20. It is generally compatible to the goals |  |
| **(9)** **Social influences (Norms)** | 21. Some members of health care team are uncomfortable watching and waiting |  |
|  | 22. Other professionals (for example: physicians, surgeons, nurses, residents, fellows ) do not influence me |  |
|  | 23. Other professionals do (for example: clinicians, nurses, physiotherapists, hematologists, blood back staff, non-ICU staff) influence me |  |
|  | 24. There is very little disagreement within my health care team |  |
|  | 25. Patients and family issue influence my practice (for example: Jehovah) |  |
| **(11)**  **Behavioural regulation** | 26. Alternatives to transfusing include prescribing vitamins, iron, EPO, nutritional support and taking less blood for testing. |  |
|  | 27. Widely accepted Protocols or Guidelines or Standard of practice |  |
|  | 28. Processes to educate health care team |  |
|  | 29. Increasing team communication |  |
|  | 30. Strong evidence |  |
|  | 31. Audit and feedback |  |
