# Supplementary material for: A cross-country comparison of intensive care physicians’ beliefs about their transfusion behaviour: A qualitative study using the theoretical domains framework
Source: Implement Sci. 2012 Sep 21;7:93. doi: 10.1186/1748-5908-7-93 (PMC3527303; doi:10.1186/1748-5908-7-93)
Supplement: Additional file 3 — Domains. [file 1748-5908-7-93-S3.docx]

**Additional File 3:**

**Domains Constructs**

Knowledge Knowledge

Knowledge about condition/scientific rationale

Schemas + mindsets + illness representations

Procedural knowledge

Social/professional Identity

Role and identity Professional identity/boundaries/role

(self-standards) Group/social identity

Social/group norms

Alienation/organizational commitment

Beliefs about Self-efficacy

Capabilities Control-of behaviour and material and social environment

Perceived competence

Self-confidence/professional confidence

Empowerment

Self-esteem

Perceived behavioural control

Optimism/pessimism

Beliefs about Outcome expectations

Consequences Anticipated regret

Appraisal/evaluation/review

Consequents

Attitudes

Contingencies

Reinforcement/punishment/consequences

Incentives/rewards

Beliefs

Unrealistic optimism

Salient events/sensitisation/critical incidents

Characteristics of outcome expectancies - physical, social, emotional;

Sanctions/rewards, proximal/distal,

Valued/not valued, probable/improbable, salient/not salient,

perceived risk/threat

Motivation and goals Intention; stability of intention/certainty of intention

Goals (autonomous, controlled)

Goal/target setting

Goal priority

Intrinsic motivation

Commitment

Distal and proximal goals

Transtheoretical model and stages of change

Social influences Social support

(norms) Social/group norms

Organizational development

Leadership

Team working

Group conformity

Organizational climate/culture

Social pressure

Power/hierarchy

Professional boundaries/roles

Management commitment

Supervision

Inter-group conflict

Champions

Social comparisons

Identity; group/social identity

Organizational commitment/alienation

Feedback

Conflict – competing demands, conflicting roles

Change management

Crew resource management

Negotiation

Social support: personal/professional/organizations, intra/interpersonal, society/community

Social/group norms: subjective, descriptive, injunctive norms

Learning and modeling

Behavioural Goal/target setting

Regulation Implementation intention

Action planning

Self-monitoring

Goal priority

Generating alternatives

Feedback

Moderators of intention-behaviour gap

Project management

Barriers and facilitators
